# Evaluating Effectiveness and Safety in Chronic Kidney Disease with Atrial Flutter Using an Anticoagulation Strategy

**DOI:** 10.3390/medicina56060266

**Published:** 2020-05-28

**Authors:** Ying-Ting Wang, Chung-Yu Chen, Ming-Jong Bair

**Affiliations:** 1 Master Program in Clinical Pharmacy, School of Pharmacy, Kaohsiung Medical University, Kaohsiung 80708, Taiwan; v125697@gmail.com; 2 Department of Pharmacy, Kaohsiung Chang Gung Memorial Hospital, Kaohsiung 833, Taiwan; 3 Department of Pharmacy, Kaohsiung Medical University Hospital, Kaohsiung 80708, Taiwan; 4 Department of Medical Research, Kaohsiung Medical University Hospital, Kaohsiung 80708, Taiwan; 5 Division of Gastroenterology, Department of Internal Medicine, Taitung MacKay Memorial Hospital, Taitung 95054, Taiwan; 6 MacKay Medical College, New Taipei City 252, Taiwan

**Keywords:** oral anticoagulants, oral antiplatelet, atrial flutter, chronic kidney disease, cardiovascular events

## Abstract

*Background and objectives:* Recent randomized trials of oral antithrombotic drugs with atrial flutter (AFL) excluded patients with renal impairment because of their increased risk of bleeding. To date, no relevant studies have assessed the effectiveness and safety of different antithrombotic drugs in chronic kidney disease (CKD) patients with AFL. This cohort study evaluated the effectiveness and safety of different antithrombotic drugs in CKD patients with AFL. This study also investigated the risk of cardiovascular events from antithrombotic drugs through different risk profiles of stroke stratified by the CHA_2_DS_2_-VASc score. *Materials and Methods:* This cohort study was performed in patients with AFL and CKD who were extracted from the National Health Insurance (NHI) Database in Taiwan. Oral antithrombotic therapy (oral anticoagulants (OAC) or antiplatelets (APT)) was administered to patients who had been diagnosed with AFL after being diagnosed with CKD between 2011 and 2015. Primary outcomes, including ischemic stroke, systemic embolism, and composite of stroke, and secondary outcomes, including major adverse cardiac events (MACEs), major bleeding, all-cause mortality, and cardiovascular-related death, were examined. *Results*: A total of 2468 patients were included in this study. The results showed no statistically significant differences in the risk of primary outcomes. For the secondary outcomes, there were also no statistically significant differences in the risk of MACEs and major bleeding. However, the pooled results indicated that the hazard ratio (HR) for all-cause mortality with OAC was 0.24 (95% confidence interval (CI) = 0.10–0.55) compared with combination therapy, and the HR for APT compared with OAC was 2.86 (95% CI = 1.48–5.53). *Conclusions:* In the studied population, OAC or APT alone were proved equally effective for stroke prophylaxis. Furthermore, OAC might reduce the all-cause mortality rate compared with APT and should be considered as the first choice of oral antithrombotic drugs in patients with AFL and CKD.

## 1. Introduction

Chronic kidney disease (CKD) is characterized by the abnormal structure or function of the kidney. The prevalence of CKD stages 1–5 is approximately 11.9% in Taiwan. [1,2] To date, some studies have reported an association between CKD and ischemic stroke. [3,4] Antithrombotic therapies (including antiplatelets (APT) and oral anticoagulants (OAC)) are commonly used in patients with CKD to treat ischemic stroke and systemic embolism of atrial fibrillation (AF) [5]. However, no studies have assessed the clinical outcomes in patients with atrial flutter (AFL) and CKD. Oral antithrombotic therapies are recommended for patients with AFL and CKD in clinical practice on the basis of the treatment recommended for use in patients with AF. However, some previous studies have shown that the risk of ischemic stroke differs between the two atrial arrhythmias, AF and AFL [6,7]. Therefore, the risk of ischemic stroke in patients with AFL should be re-evaluated.

Additionally, recent studies showed increased resistance to APT (aspirin and clopidogrel) in patients with kidney impairment compared with patients without CKD [8,9,10]. Therefore, the protective effect of APT may be reduced in patients with CKD, particularly in those with end-stage renal disease undergoing renal replacement [11,12]. Conversely, OAC (warfarin and direct oral anticoagulants) are commonly recommended in patients with AF and CKD. However, impaired renal function massively increases the risk of hemorrhage in patients taking OAC [13]. Moreover, randomized trials on the benefits of anticoagulation on patients with CKD, particularly with advanced CKD (estimated glomerular filtration rate (GFR) <30 mL/min/1.73 m^2^), are lacking. Therefore, the clinical outcomes of the use of different antithrombotic drugs must be evaluated in patients with renal impairment.

However, no clinical trials or observational studies have evaluated the effectiveness and safety of different antithrombotic drugs for stroke prevention in patients with AFL and CKD. Thus, the effect of oral antithrombotic therapies (APT and OAC), which are usually used for stroke prophylaxis, is uncertain in this population. To address this concern, this study investigated the effectiveness and safety of different types of oral antithrombotic drugs for cardiovascular-related diseases in patients with AFL and CKD who were identified through the National Health Insurance (NHI) Database in Taiwan.

## 2. Materials and Methods

### 2.1. Data Sources

This retrospective cohort study utilized data from the NHI Database in Taiwan, which was established in 1995. This system covers 99% of the 23 million Taiwan individuals under compulsory health insurance. The healthcare information in the database includes outpatients, hospitalizations, ambulatory services, dental services, and prescription drugs. Furthermore, this cohort study used the full insurance coverage data in Taiwan from 1 January 2010 to 31 December 2017. The International Classification of Diseases, Ninth Revision, Clinical Modification (ICD-9-CM) and the International Classification of Diseases, Tenth Revision, Clinical Modification (ICD-10-CM) were used to extract disease information from the database. AFL (ICD-9-CM: 427.32), CKD (ICD-9-CM: 250.4, 572.4, 403, 404, 581–583, 585–588), and all comorbidities were defined as present when a diagnosis was made at least once during hospitalization or on two consecutive clinical visits.

This study was approved by the Institutional Review Board of Kaohsiung Medical University Hospital, Kaohsiung, Taiwan. The Institutional Review Board number was KMUHIRB-EXEMPT (I)-20190009, and the approved date was 2019/03/05. This study was performed in accordance with the ethical standards established in the 1964 Declaration of Helsinki and its later amendments.

### 2.2. Study Sample

All patients who were diagnosed with AFL after being diagnosed with CKD between 1 January 2011 and 31 December 2015 were included. The first date that a patient was prescribed an oral antithrombotic drug after the AFL diagnosis date was designated as the index date. Patients who were younger than 20 years old at the date of the first AFL episode during the study period and those who had missing data were excluded. Furthermore, patients who were diagnosed with AF, cirrhosis, mitral stenosis, or stroke, and those who had received renal transplantation or mechanical valves before the date of the first AFL episode during the study period were also excluded. The definition of desired outcomes for this study according to the ICD-9-CM and ICD-10-CM codes are presented in Online Table S1. Other definitions of disease according to the NHI code, the ICD-9-PCS, and the ICD-10-PCS are presented in Online Table S2. Comorbidities including chronic obstructive pulmonary disease, dyslipidemia, gout, abnormal liver function, and gastric ulcer were defined as present when there were at least two outpatient or one inpatient diagnosis code within one year before the index date. The use of concomitant drugs, including non-steroidal anti-inflammatory drugs, proton pump inhibitors, H2-blockers, angiotensin converting enzyme inhibitors/angiotensin receptor blockers, calcium channel blockers, beta blockers, statins, digoxin, and amiodarone, was considered when the patients had received at least two prescriptions within one year before the index date. Details on the concomitant drugs according to their ATC codes are listed in Online Table S3.

### 2.3. Medication Use

**Figure 1:**
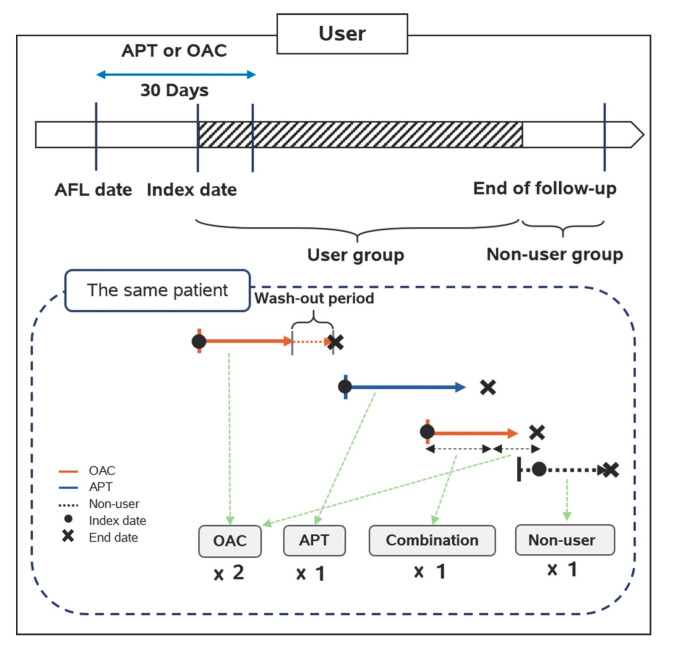
Drug exposure definition in the cohort study. OAC: oral anticoagulants; APT: antiplatelets; AFL: atrial flutter.

Oral antithrombotic drugs included oral APT and OAC. The definition of combination therapy in this study included the following three regimens: (1) dual APT, (2) an APT combined with an OAC, and (3) dual APT combined with an OAC. This study used NIH codes to identify target drugs in the database. The NIH codes of the drugs are presented in Online Table S4. The detailed drug information included in this cohort study is shown in Online Table S5. Patients who took any antithrombotic drug, e.g., APT or OAC, within 30 days after the AFL diagnosis date were classified into the APT group and the OAC group, respectively. The patients who were eligible for inclusion in a user group were continuously followed until the day of the desired outcome or censored events. If medication-transferring occurred, the patient was transferred to the latter group and followed up on. The wash-out period between the two different groups was assumed to be seven days. In addition, patients in the user group were transferred to the non-user group and continuously followed-up when they discontinued their medication. (Figure 1).

### 2.4. Outcomes

The primary outcome in this study was patient admission to the emergency room or hospitalization due to ischemic stroke, systemic embolism, and composite of stroke. The secondary outcomes included major adverse cardiac events (MACEs), major bleeding, all-cause mortality, and cardiovascular-related death. A MACE was defined as an emergency department visit with hospitalization for reasons including acute myocardial infarction, ischemic stroke, systemic embolism, coronary artery bypass grafting, percutaneous coronary intervention, and cardiovascular-related death. Furthermore, cardiovascular-related death included cardiac-related death, cerebrovascular-related death, and atherosclerosis-related death. Each patient was followed-up for at least two years until the day of the desired outcome or censored events. Patients who did not experience the primary or secondary outcomes were censored in this study. The definition of censored events in the user group included death, AF, or the end date of the study period.

### 2.5. Statistics

Differences in baseline characteristics were evaluated using a one-way ANOVA for continuous variables (e.g., age) or the chi-squared test for categorical variables (e.g., gender, CHA_2_DS_2_-VASc score [congestive heart failure, hypertension, age (≥75 years; 2 points), diabetes, stroke/transient ischemic attack (2 points), vascular disease, age (65–74 years), 1 points in males and 2 points in females], HAS-BLED score [hypertension, abnormal renal/liver function, stroke, bleeding history or predisposition, labile INR, elderly, drugs/alcohol concomitantly, 1 points in each variable], comorbidities, and concomitant drugs). This study calculated the incidence of the events (unit: events/1000 person-years) by dividing the number of the events by the total person-years of follow-up. Furthermore, to consider other potential confounders, Cox regression hazard models were performed to calculate the hazard ratios (HRs) and 95% confidence intervals (CIs). Both univariate and multivariable analyses were performed. For the statistical analyses in this study, *p* < 0.05 was considered statistically significant. To evaluate clinical outcomes among patients with the same stroke risk, subgroup analyses were performed to separate the risk according to the CHA2DS2-VASc score. Otherwise, gender was an important factor for risk of ischemic stroke, and subgroup analyses were performed to separate gender to evaluate the risk of ischemic stroke and all-cause mortality. Furthermore, the study also created a receiver operating characteristic (ROC) curve to explore the cutoff point to stratify the event risk according to the CHA_2_DS_2_-VASc score. All the analyses were performed using SAS statistical software (Version 9.4; SAS Institute, Inc., Cary, NC, USA).

## 3. Results

### 3.1. Study Population Included in the Study

**Figure 2:**
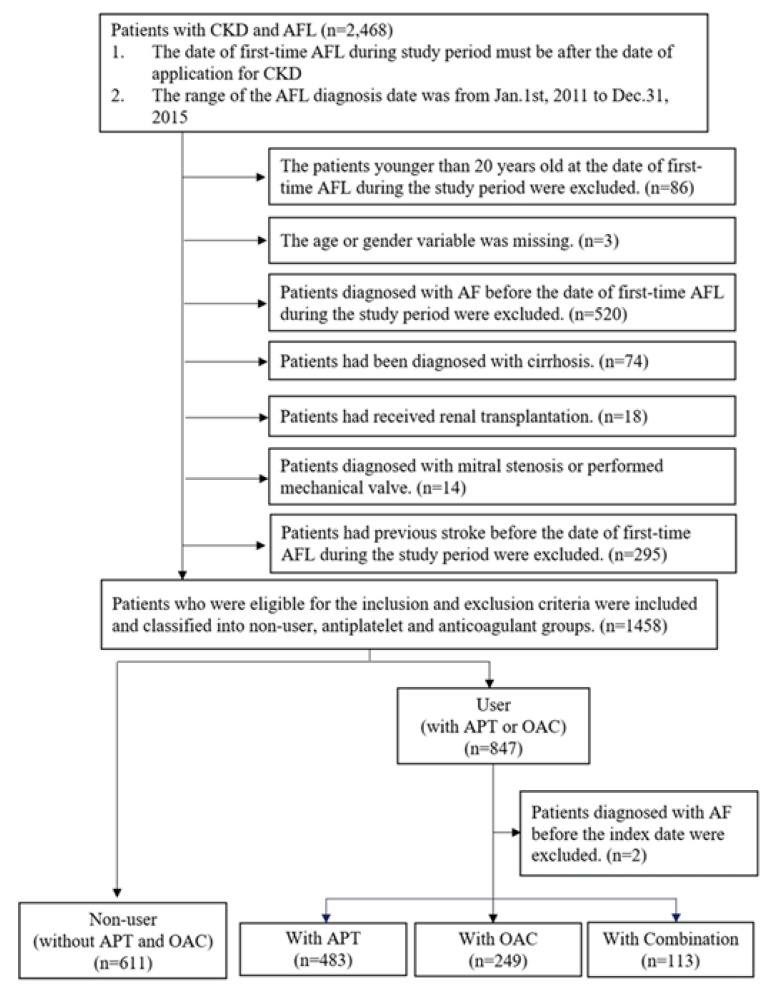
Results of the study population selection. AF: atrial fibrillation; CKD: chronic kidney

In total, 2468 patients with CKD and AFL were identified from the data sources, and 89 patients who were younger than 20 years old at the date of the first AFL episode during the study period as well as those with missing data were excluded. Additionally, 921 patients who were diagnosed with AF, cirrhosis, mitral stenosis, or stroke, or those who received renal transplantation or mechanical valves before the date of the first AFL episode during the study period were excluded. A total of 611 and 847 patients were classified into the non-user and user groups, respectively. Moreover, two patients who were diagnosed with AF before the index date were excluded from the user group. Finally, 483, 249, and 113 patients were prescribed APT, OAC, and combination therapy in the user group, respectively (Figure 2).

### 3.2. Demographic Characteristics of Study Populations

The mean age of the population was approximately 70.82 years old. No significant differences in age were noted among all the therapies (mean age in APT: 70.78 years; mean age in OAC: 71.30 years; mean age in combination therapy: 69.94 years; *p* = 0.557) (Table 1). There was a higher proportion of males than females, and the male-to-female ratio was 2.5:1 in the overall population. In the evaluation of comorbidities, the combination group had significantly higher prevalence rates of diabetes mellitus (*p* = 0.044) and vascular disease (*p* < 0.001). No significant differences were found among the three groups for the distributions of comorbidities such as congested heart failure, hypertension, chronic obstructive pulmonary disease, dyslipidemia, gout, abnormal liver function, gastric ulcer, and bleeding history. When evaluating concomitant drug use, patients in the combination group had significantly higher prescription rates for hypertension drugs such as beta-blockers (*p* = 0.006), calcium channel blockers (*p* = 0.006), amiodarone (*p* = 0.005), statins (*p* < 0.001), non-steroidal anti-inflammatory drugs (*p* = 0.044), and proton pump inhibitors (*p* < 0.001). Nevertheless, there were no significant differences among the groups in the distributions of angiotensin-converting enzyme inhibitors/angiotensin receptor blockers, digoxin, and H2-blockers. Furthermore, the mean CHA_2_DS_2_-VASc score in the population was close to 3. The mean score of the combination group was significantly higher than that of the other groups (mean score: 3.26; standard deviation: 1.27; *p* = 0.004). The mean HAS-BLED score in the population was approximately 3.19. According to the HAS-BLED score, the risk of bleeding was higher in the APT group than in the other two groups (mean score: 3.51; standard deviation: 0.80; *p* < 0.001). At combination group, there were 56 patents who ever used dual antiplatelet, 52 patients who ever used APT combined with an OAC, and only 5 patients who ever used triple therapy at follow-up time.

### 3.3. Outcome Analysis of Study Population

The incidence rates of ischemic stroke in patients receiving APT, OAC, and combination therapy were 42.88/1000 person-years, 42.26/1000 person-years, and 81.08/1000 person-years, respectively (Online Table S6). However, there was no significant difference in the risk of ischemic stroke among the APT, OAC, and combination groups according to the multivariable analysis (Table 2). The incidence rate of systemic embolism in patients receiving APT, OAC, and combination therapy was 2.46/1000 person-years, 4.46/1000 person-years, and 25.91/1000 person-years, respectively. According to the Cox regression hazard models, there was no significant difference in the risk of systemic embolism among the three groups. Furthermore, the incidence rates of composite of stroke in patients receiving APT, OAC, and combination therapy were 40.61/1000 person-years, 47.04/1000 person-years, and 109.84/1000 person-years, respectively. However, the risk of composite of stroke was similar among all the oral anticoagulation regimens according to the multivariable analysis. There were also no significant differences in the risks of the secondary outcomes at the endpoint, including MACEs and major bleeding, among the groups. Furthermore, decreases in the risks of all-cause mortality (OAC vs. combination: adjusted HR = 0.24, 95% CI = 0.10–0.55, *p* = 0.001; APT vs. OAC: adjusted HR = 2.86, 95% CI = 1.48–5.53, *p* = 0.002) and cardiovascular-related death (OAC vs. combination: adjusted HR = 0.24, 95% CI = 0.08–0.73, *p* = 0.012; APT vs. OAC: adjusted HR = 2.60, 95% CI = 1.06–6.36, *p* = 0.037) were observed in patients receiving OAC compared with patients receiving APT and combination therapy.

### 3.4. Stroke risk Stratified with CHA_2_DS_2_-VASc Score and Gender

After performing the subgroup analysis to divide the CHA_2_DS_2_-VASc score into groups with scores higher or lower than 5, there was no statistically significant difference in the risk of ischemic stroke and composite of stroke among all the therapies in the multivariable analysis. Furthermore, there was no significant difference in the risk of systemic embolism between APT and combination therapy when the score was 5 or higher (Table 3). For the secondary outcomes, there was no significant difference in the risk of MACEs and major bleeding among all the anticoagulation therapies. The subgroup analysis indicated that the OAC group had a significant lower risk of all-cause mortality compared with the other groups when the CHA_2_DS_2_-VASc score was less than 5 (OAC vs. combination: adjusted HR = 0.32, 95% CI = 0.13–0.78, *p* = 0.012; APT vs. OAC: adjusted HR = 2.15, 95% CI = 1.08–4.25, *p* = 0.029). Additionally, the OAC group had a lower cardiovascular-related mortality rate compared with the combination therapy group in the multivariable analysis when the CHA_2_DS_2_-VASc score was less than 5 (OAC vs. combination: adjusted HR = 0.30, 95% CI = 0.09–0.99, *p* = 0.048). As presented in Online Table S7, males and females showed the same trend of risk of ischemic stroke and all-cause mortality by comparing APT, OAC, and combination.

**Figure 3:**
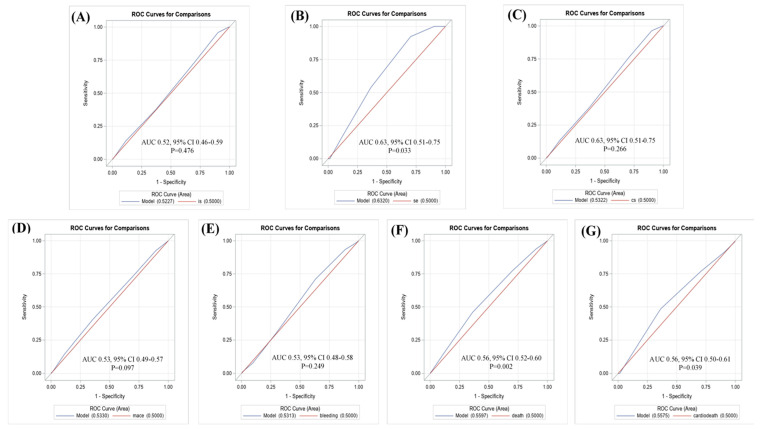
Receiver operating characteristic (ROC) curves for the CHA_2_DS_2_-VASc score for prediction of (**A**) ischemic stroke; (**B**) systemic embolism; (**C**) composite of stroke; (**D**) MACE; (**E**) major bleeding; (**F**) all-cause mortality; (**G**) cardiovascular-related death.

We also created ROC curves to identify the cutoff point to distinguish the event risk. Most of the outcomes showed that the CHA_2_DS_2_-VASc score was not a significant predictor, except for systemic embolism (area under the curve (AUC) = 0.53; 95% CI = 0.51–0.75; *p* = 0.033), all-cause mortality (AUC = 0.56; 95% CI = 0.52–0.60; *p* = 0.002), and cardiovascular-related death (AUC = 0.56; 95% CI = 0.50–0.61; *p* = 0.039) (Figure 3).

## 4. Discussion

Renal impairment causes unstable pharmacodynamics of oral antithrombotic drugs. Thus, this retrospective cohort study aimed to evaluate the effectiveness and safety among APT, OAC, and combination therapy for stroke prophylaxis in patients with AFL and CKD.

### 4.1. Effectiveness and Safety of Different Oral Antithrombotic Regimens for Stroke Prevention

There were no significant differences in the primary outcomes (ischemic stroke, systemic embolism, and composite of stroke) among all the antithrombotic regimens in patients with AFL and CKD. The combination therapy, especially in dual antiplatelet therapy and APT with OAC, was not associated with a lower rate of ischemic stroke or systemic embolism either because the additional protection afforded by APT against thromboembolism was insufficient to overcome this higher intrinsic thrombosis risk or because of some other property of APT that increased the risk of ischemic events during concurrent therapy with an OAC or dual APT. Similarly, some previous studies focusing on APT with an OAC reported the same results as this cohort study, in which combination therapy did not reduce ischemic stroke risk compared with mono OAC therapy. [14,15,16,17]. Otherwise, there was an indirect network meta-analysis that showed that dual APT only had slight benefits in ischemic stroke when compared with mono antiplatelet, and APT with OAC also had similar benefits with dual or mono APT [18]. However, a systematic review by Wong et al. found that dual APT significantly reduced the risk of stroke recurrence (relative risk = 0.69; 95% CI = 0.60–0.80; *p* < 0.001) compared with mono APT. [19] Although there was a similar trend of risk between the combination of dual antiplatelet therapy or APT with OAC, and OAC or APT in this study, there was no statistical difference in the risk of recurrent stroke between the two oral antithrombotic regimens. This discrepancy might be explained by the following reasons: (1) the population was not the same, and (2) combination therapy in the study included both dual APT and at least one APT and an OAC.

In addition, recent studies have shown that the protective effect of APT may be reduced in patients with CKD due to resistance to APT in patients with kidney impairment compared with patients without CKD [8,9,10]. However, this cohort study reported that APT had a similar risk as that of OAC for ischemic stroke, systemic embolism, and composite of stroke. Therefore, on the basis of these results, APT was not associated with a reduced effectiveness of stroke prophylaxis due to resistance to APT in patients with AFL and renal impairment. This cohort study suggests that APT could be an alternative to OAC for reducing the risk of ischemic stroke and systemic embolism in this particular population.

The results showed that OAC/APT and combined therapy had a similar risk of MACEs because the additional protection against thromboembolism by APT was insufficient to overcome the higher intrinsic thrombosis risk. This result was the same as previous studies [14,20]. However, due to the current study design, the prescription pattern of each patient changed on the basis of his/her comorbidities and disease severity. Therefore, to consider the effectiveness in preventing MACEs, clinicians should prescribe different oral antithrombotic regimens to reduce the incidence rate of MACEs on the basis of the particular clinical scenario.

For major bleeding, combination therapy should induce excessive inhibitors for coagulation and the fibrinolytic pathway and increase the hemorrhage events on the basis of pharmacodynamics. However, the Cox regression hazard model in this study did not show any differences between OAC/APT and combination therapy in terms of major bleeding. A systematic review also showed that combination therapy and OAC/APT had similar risks of major bleeding [21]. Conversely, another systematic review by Dentali et al. reported that the risk of major bleeding was higher in patients receiving combination therapy compared with patients receiving single therapy (odds ratio = 1.43; 95% CI = 1.00–2.02) [14]. Discrepancy between the results from this cohort study and the previous study might be explained by the different populations and differences in the definition of combination therapy between the studies.

A previous meta-analysis indicated that OAC had no significant differences in all-cause mortality compared with APT with OAC [14]. However, this study showed that OAC had a lower all-cause mortality rate (OAC vs. combination (dual APT/APT with OAC): adjusted HR = 0.24, 95% CI = 0.10–0.55, *p* = 0.001; APT vs. OAC: adjusted HR = 2.86, 95% CI = 1.48–5.53, *p* = 0.002). The discrepancy between the two studies might be explained by the following reasons: (1) the race of the populations was not the same (the meta-analysis was performed in a non-Asian region; however, this cohort study was performed in an Asian region); (2) the quality of the randomized controlled trials included in the meta-analysis was not consistent; and (3) some studies included in the meta-analysis had populations with mechanical valves, which was excluded in this cohort study. Thus, OAC might have the potential to reduce the mortality rate compared with APT and combination therapy. Nevertheless, the mortality risk must continue to be evaluated among the different antithrombotic regimens in future studies.

### 4.2. Subgroup Analysis to Stratify Event Risk by CHA_2_DS_2_-VASc Score and Gender

A previous study by Lin et al. [22] recommended the use of oral anticoagulation therapy for patients with AFL when the CHA_2_DS_2_-VASc score was 5 or higher. The results indicated that both OAC and APT had safety and effectiveness similar to that of combination therapy. Additionally, OAC might have a lower mortality rate, both for all-cause mortality (OAC vs. combination: adjusted HR = 0.32, 95% CI = 0.13–0.78, *p* = 0.012) and cardiovascular-related death (OAC vs. combination: adjusted HR = 0.30, 95% CI = 0.09–0.99, *p* = 0.048), compared to combination therapy when the CHA2DS2-VASc score is less than 5. However, further studies must evaluate the mortality risk stratified by the CHA_2_DS_2_-VASc score between OAC and combination therapy in the future.

From CHA_2_DS_2_-VASc score, females had a higher risk of stroke in AF than males [23]. However, there is limited information in AFL patients. Furthermore, the effectiveness of different antithrombotic drugs for stroke prevention in women versus men with AF is still poorly known [22]. This study’s results showed that there were no significant impact levels on females or males using different antithrombotic drugs, which produced the same result as previous meta-analyses and evidence [24,25,26].

Furthermore, the ROC curve was used to explore the CHA_2_DS_2_-VASc score cutoff point that can be used to distinguish event risk. The results showed that the CHA_2_DS_2_-VASc score was a significant predictor of systemic embolism (AUC = 0.53; 95% CI = 0.51–0.75; *p* = 0.033), all-cause mortality (AUC = 0.56; 95% CI = 0.52–0.60; *p* = 0.002), and cardiovascular-related death (AUC = 0.56; 95% CI = 0.50–0.61; *p* = 0.039), and could be used to distinguish the event risk. However, all the AUC values were closer to 50%, which indicated that the CHA_2_DS_2_-VASc score is not an appropriate assessment tool for stratifying event risk in AFL and CKD. Therefore, future studies should reevaluate the assessment score that is used to predict the risk of stroke or mortality in patients with AFL.

### 4.3. Study Strengths and Limitations

Important strengths of this study include the sample size and the study design that addressed the issue of medication-transferring. To our knowledge, this study is the first to evaluate the risk of cardiovascular events in CKD patients with AFL among different oral antithrombotic regimens. The study was conducted using the entire population database of Taiwan to evaluate clinical outcomes. This database covers 99% of the 23 million Taiwan individuals under compulsory health insurance. Moreover, this study solved the problem of medication-transferring, which is a common problem in observational studies that evaluate the short-term effects of drugs.

Some limitations of this study should be mentioned. First, some variables, such as the international normalized ratio (INR) value and alcohol drinking habits, were not obtained from the NHI Database in Taiwan. Therefore, alcohol drinking habits were only estimated using relevant diagnoses, e.g., alcohol liver damage and alcohol use disorder, in the ICD-9-CM. For these reasons, the HAS-BLED score may have been underestimated in this study. Second, the GFR value could not be obtained from the NHI Database to distinguish the stage of CKD. Thus, randomized controlled trials are necessary to explore the clinical outcomes on the basis of different CKD stages. Third, the number of hospital days was used to indicate the number of days a patient was on a medication because the database only contained information on expenditures from hospital admissions, which would cause overestimated the medication exposure. Fourth, for the combination group, there were only five patients who used triple therapy at the follow-up time, and thus it was hard to investigate the outcome of triple therapy than other mono or dual therapy. Finally, the NHI Database does not contain information from clinical physical examinations, such as body weight, blood pressure, and low-density lipoprotein cholesterol level, which are associated with the risk of ischemic stroke [27]. Despite these limitations, this study provides information on the use of different oral antithrombotic drugs in patients with CKD and AFL. The results reported that there was no significant risk of cardiovascular events among oral APT, OAC, and combination therapy regimens. Future randomized controlled trials are necessary to evaluate the effectiveness and safety of different anticoagulation therapies in patients with AFL and CKD.

## 5. Conclusions

In conclusion, this study reported that there was no statistically significant differences in the risk of ischemic stroke or systemic embolism among oral APT, OAC, and combination therapy in patients with CKD and AFL. Therefore, in the studied population OAC or APT alone were proved equally effective for stroke prophylaxis in patients with AFL and renal impairment. Furthermore, OAC might reduce the all-cause mortality rate compared with oral APT and should be considered as the first choice of oral antithrombotic drugs in patients with AFL and CKD. In the future, randomized controlled trials are necessary to evaluate the efficacy and safety for stroke prevention in patients with CKD and AFL using different oral antithrombotic drugs. Finally, whether the CHA_2_DS_2_-VASc score can be used to assess the risk of ischemic stroke or mortality in patients with CKD and AFL should be re-evaluated in the future.

## Figures and Tables

**Table 1:** Baseline characteristics of antiplatelets (APT), oral anticoagulants (OAC), and combination group.

Characteristics	APT	OAC	Combination	*p*-Value
	*n* = 483	*n* = 249	*n* = 113	
**Age, mean (SD), years**	70.78 (11.29)	71.30 (10.91)	69.94 (11.02)	0.557
Age group (%)				
<65	135 (27.95)	57 (22.89)	34 (30.09)	0.874
65–74	144 (29.81)	72 (28.92)	37 (32.74)	
≥75	204 (42.24)	109 (43.78)	42 (37.17)	
Gender (%)				
Male	330 (68.32)	185 (74.30)	89 (78.76)	0.044
Female	153 (31.68)	64 (25.70)	24 (21.24)	
Comorbidity (%)				
Congestive heart failure	109 (22.57)	54 (21.69)	33 (29.20)	0.257
Hypertension	294 (60.87)	140 (56.22)	71 (62.83)	0.371
Diabetes mellitus	219 (45.34)	92 (36.95)	55 (48.67)	0.044
Vascular disease	35 (7.25)	3 (1.20)	34 (30.09)	<0.001
Chronic obstructive pulmonary disease	39 (8.07)	17 (6.83)	12 (10.62)	0.470
Dyslipidemia	87 (18.01)	43 (17.27)	27 (23.89)	0.287
Gout	59 (12.22)	28 (11.24)	10 (8.85)	0.595
Abnormal liver function	53 (10.97)	19 (7.63)	6 (5.31)	0.101
Gastric ulcer	50 (10.35)	19 (7.63)	10 (8.85)	0.479
Bleeding history	50 (10.35)	21 (8.43)	11 (9.73)	0.708
Concomitant drugs (%)				
Beta blocker	287 (59.42)	169 (67.87)	83 (73.45)	0.006
CCB	297 (61.49)	124 (49.80)	71 (62.83)	0.006
ACEI/ARB	214 (44.31)	126 (50.60)	63 (55.75)	0.050
Amiodarone	192 (39.75)	104 (41.77)	64 (56.64)	0.005
Statin	155 (32.09)	71 (28.51)	59 (52.21)	<0.001
Digoxin	69 (14.29)	48 (19.28)	20 (17.70)	0.199
NSAID	306 (63.35)	140 (56.22)	78 (69.03)	0.044
PPI	80 (16.56)	22 (8.84)	33 (29.20)	<0.001
H_2_-blocker	104 (21.53)	45 (18.07)	33 (29.20)	0.058
CHA_2_DS_2_-VASc score, mean (SD)	3.05 (1.29)	2.82 (1.09)	3.26 (1.27)	0.004
HAS-BLED score, mean (SD)	3.51 (0.80)	2.44 (0.75)	3.45 (0.73)	<0.001

APT, antiplatelet; OAC, oral anticoagulant; SD, standard deviation; CCB, calcium channel blocker; ACEI, angiotensin-converting-enzyme inhibitor; ARB, angiotensin II receptor antagonists; NSAID, non-steroidal anti-inflammatory; PPI, proton pump inhibitors; CHA_2_DS_2_-VASc score = congestive heart failure, hypertension, age 75 years or older, diabetes mellitus, age 65 to 74 years, female, previous stroke, and vascular disease (prior myocardial infarction and peripheral artery disease); HAS-BLED score = hypertension, age 65 years or older, alcohol-related history, drugs (NSAID or APT), previous stroke, abnormal liver function, abnormal kidney function, and bleeding history.

**Table 2:** Cox-regression hazard models compared among the APT, OAC, and combination groups.

	Crude Hazard Ratio						Adjusted Hazard Ratio					
	APT vs. Combination		OAC vs. Combination		APT vs. OAC		APT vs. Combination		OAC vs. Combination		APT vs. OAC	
	95% CI	*p*-Value	95% CI	*p*-Value	95% CI	*p*-Value	95% CI	*p*-value	95% CI	*p*-Value	95% CI	*p*-Value
Ischemic stroke	0.61 (0.24–1.57)	0.307	0.66 (0.23–1.91)	0.448	1.04 (0.46–2.34)	0.924	0.54 (0.21–1.44)	0.222	0.58 (0.18–1.87)	0.361	1.07 (0.47–2.46)	0.873
Systemic embolism	0.09 (0.01–1.10)	0.059	0.26 (0.02–2.95)	0.277	0.55 (0.03–8.80)	0.672	0.06 (0.00–1.67)	0.097	0.29 (0.02–3.65)	0.335	0.33 (0.02–6.68)	0.467
Composite of stroke	0.42 (0.18–1.00)	0.051	0.56 (0.22–1.45)	0.235	0.89 (0.40–1.95)	0.761	0.42 (0.18–1.01)	0.051	0.63 (0.22–1.82)	0.390	0.91 (0.40–2.06)	0.819
MACE	0.89 (0.45–1.79)	0.747	0.64 (0.29–1.41)	0.269	1.40 (0.81–2.42)	0.234	0.93 (0.46–1.89)	0.850	0.71 (0.30–1.67)	0.436	1.34 (0.76–2.36)	0.315
Major bleeding	0.93 (0.31–2.75)	0.895	1.44 (0.46–4.47)	0.533	0.86 (0.44–1.68)	0.652	0.97 (0.33–2.87)	0.957	1.19 (0.37–3.84)	0.775	0.91 (0.46–1.80)	0.779
All-cause mortality	0.73 (0.42–1.27)	0.266	0.22 (0.10–0.49)	<0.001	2.88 (1.51–5.49)	0.001	0.86 (0.49–1.51)	0.596	0.24 (0.10–0.55)	0.001	2.86 (1.48–5.53)	0.002
Cardiovascular-related death	0.69 (0.34–1.42)	0.318	0.23 (0.08–0.64)	0.005	2.88 (1.20–6.90)	0.018	0.79 (0.38–1.66)	0.530	0.24 (0.08–0.73)	0.012	2.60 (1.06–6.36)	0.037

APT, antiplatelet; OAC, oral anticoagulants; MACE, major adverse cardiac event; CI, confidential interval. Adjusted variables included age, gender, congested heat failure, hypertension, diabetes mellitus, vascular diseases, chronic obstructive pulmonary disease, dyslipidemia, gout, abnormal liver function, gastric ulcer, and bleeding history.

**Table 3:** Subgroup analysis stratified with the CHA_2_DS_2_-VASc score in patients with atrial flutter (AFL) and chronic kidney disease (CKD).

	Crude Hazard Ratio						Adjusted Hazard Ratio					
	APT vs. Combination		OAC vs. Combination		APT vs. OAC		APT vs. Combination		OAC vs. Combination		APT vs. OAC	
Subgroup	95% CI	*p*-Value	95% CI	*p*-Value	95% CI	*p*-Value	95% CI	*p*-Value	95% CI	*p*-Value	95% CI	*p*-Value
Ischemic stroke												
CHA_2_DS_2_-VASc												
<5	0.81 (0.27–2.48)	0.713	0.85 (0.25–2.89)	0.794	1.07 (0.46–2.54)	0.870	0.78 (0.25–2.44)	0.665	0.93 (0.22–3.95)	0.926	1.12 (0.46–2.74)	0.799
≥5	0.20 (0.02–1.66)	0.135	0.35 (0.03–3.90)	0.390	0.84 (0.08–9.32)	0.890	0.19 (0.02–1.72)	0.139	0.31 (0.03–3.66)	0.353	0.81 (0.05–14.34)	0.886
Systemic embolism												
CHA_2_DS_2_-VASc												
<5	-	-	-	-	-	-	-	-	-	-	-	-
≥5	0.10 (0.01–2.04)	0.134	-	-	-	-	0.11 (0.01–2.21)	0.149	-	-	-	-
Composite of stroke												
CHA_2_DS_2_-VASc												
<5	0.60 (0.21–1.68)	0.327	0.80 (0.26–2.44)	0.693	0.89 (0.39–2.06)	0.788	0.60 (0.21–1.74)	0.349	0.98 (0.26–3.78)	0.981	0.93 (0.39–2.24)	0.876
≥5	0.11 (0.02–0.84)	0.033	0.20 (0.02–1.92)	0.161	0.87 (0.08–9.61)	0.910	0.23 (0.01–6.95)	0.399	0.19 (0.02–1.91)	0.156	0.81 (0.05–14.34)	0.886
MACE												
CHA_2_DS_2_-VASc												
<5	1.03 (0.46–2.33)	0.940	0.81 (0.33–1.98)	0.641	1.27 (0.71–2.26)	0.420	1.11 (0.49–2.55)	0.801	0.82 (0.32–2.15)	0.690	1.16 (0.64–2.11)	0.633
≥5	0.66 (0.17–2.55)	0.546	0.24 (0.02–2.29)	0.213	3.27 (0.41–26.13)	0.265	0.43 (0.10–1.90)	0.265	0.12 (0.00–6.47)	0.297	3.73 (0.39–36.03)	0.255
Major Bleeding												
CHA_2_DS_2_-VASc												
<5	1.23 (0.36–4.18)	0.740	1.82 (0.51–6.46)	0.353	0.85 (0.43–1.67)	0.627	1.27 (0.36–4.49)	0.712	1.48 (0.33–6.66)	0.614	0.88 (0.44–1.78)	0.729
≥5	-	-	-	-	-	-	-	-	-	-	-	-
All-cause mortality												
CHA_2_DS_2_-VASc												
<5	0.69 (0.37–1.26)	0.222	0.26 (0.11–0.57)	0.001	2.30 (1.19–4.45)	0.014	0.82 (0.44–1.54)	0.538	0.32 (0.13–0.78)	0.012	2.15 (1.08–4.25)	0.029
≥5	1.09 (0.31–3.90)	0.894	-	-	-	-	1.20 (0.30–4.70)	0.798	-	-	-	-
Cardiovascular-related death												
CHA_2_DS_2_-VASc												
<5	0.64 (0.30–1.37)	0.248	0.24 (0.09–0.70)	0.008	2.51 (1.03–6.10)	0.042	0.75 (0.34–1.68)	0.489	0.30 (0.09–0.99)	0.048	2.06 (0.82–5.17)	0.122
≥5	1.43 (0.17–12.36)	0.744	-	-	-	-	1.41 (0.14–14.64)	0.772	-	-	-	-

“-” means limited data; APT, antiplatelet; OAC, oral anticoagulants; MACE, major adverse cardiac event; CI, confidential interval. Adjusted variables included age, gender, congested heat failure, hypertension, diabetes mellitus, vascular diseases, chronic obstructive pulmonary disease, dyslipidemia, gout, abnormal liver function, gastric ulcer, and bleeding history.

## Supplementary Materials

The following are available online at https://www.mdpi.com/1010-660X/56/6/266/s1, Table S1. International Classification of Diseases, Ninth Revision, Clinical Modification and International Classification of Diseases, Tenth Revision, Clinical Modification used for disease extraction, Table S2. International Classification of Diseases, Ninth Revision, Procedure Coding System, International Classification of Diseases, Tenth Revision, Procedure Coding System and National Health Insurance code used for study variables, Table S3. The drug code of concomitants in the study, Table S4. The drug code of oral antithrombotic therapies in the study, Table S5. Oral antithrombotic therapies included in the study, Table S6. Incidence of study outcomes among APT, OAC and combination group, Table S7. Subgroup analysis stratified with gender in patients with AFL and CKD.
