# Supplementary material for: Evaluating Effectiveness and Safety in Chronic Kidney Disease with Atrial Flutter Using an Anticoagulation Strategy
Source: Medicina (Kaunas). 2020 May 28;56(6):266. doi: 10.3390/medicina56060266 (PMC7353883; doi:10.3390/medicina56060266)
Supplement: Supplementary file 1 [file medicina-56-00266-s001.pdf]

Supplement to:

Evaluating Effectiveness and Safety in Chronic Kidney Disease with Atrial Flutter using Anticoagulation Strategy

Ying-Ting Wang, Chung-Yu Chen, Ming-Jong Bair

Table of Contents

Supplemental Table S1. International Classification of Diseases, Ninth Revision, Clinical Modification and International Classification of Diseases, Tenth Revision, Clinical Modification used for disease extraction 2

Supplemental Table S2. International Classification of Diseases, Ninth Revision, Procedure Coding System, International Classification of Diseases, Tenth Revision, Procedure Coding System and National Health Insurance code used for study variables 5

Supplemental Table S3. The drug code of concomitants in the study 6

Supplemental Table S4. The drug code of oral antithrombotic therapies in the study 8

Supplemental Table S5. Oral antithrombotic therapies included in the study 11

Supplemental Table S6. Incidence of study outcomes among APT, OAC and combination group 12

Supplemental Table S7. Subgroup analysis stratified with gender in patients with AFL and CKD 13

Supplemental Table S1. International Classification of Diseases, Ninth Revision, Clinical Modification and International Classification of Diseases, Tenth Revision, Clinical Modification used for disease extraction

Items	ICD-9-CM	ICD-10-CM
**Inclusion/exclusion criteria**		
Atrial flutter	427.32	
Chronic kidney disease	250.4, 572.4, 403, 404, 581, 582, 583, 585, 586, 587, 588	
Cirrhosis	571.2, 571.5, 571.6	
Mitral Stenosis	394.0, 396.0	I05.0, I08.0
**Comorbidity**		
Hypertension	401, 402, 403, 404, 405	
Diabetes mellitus	250	
Congested heart failure	428	
Abnormal liver function	070, 570, 571, 573, 456.0, 456.1, 456.2, 572.2, 572.3, 572.4, 572.5, 572.6, 572.7, 572.8	
Previous stroke	433, 434, 444, 430, 431, 432, 435	
Vascular disease	410, 412, 440.2	

Items	ICD-9-CM	ICD-10-CM
Gastric ulcer	531, 532, 533, 534	
Dyslipidemia	272	
Gout	274	
Chronic obstructive pulmonary disease	491, 492, 496	
Cirrhosis	571.2, 571.5, 571.6	
Alcoholic liver damage	571.00, 571.10, 571.20, 571.30	
Alcohol use disorder	303, 305, V11.3	
**Desired outcome**		
Ischemic Stroke	433, 434, 535, 436, 437	I63, I65, I66, G45.0, G45.1, G45.2, G45.4, G45.8, G45.9, G46, I47, I67.0, I67.1, I67.2, I67.4, I67.5, I67.6, I67.7, I67.9, I67.841, I67.848, I67.81, I67.82, I67.89, I68.0, I68.2, I68.8
Systemic Embolism	444	I74
Acute Myocardial Infarction	410	I21, I22

Items	ICD-9-CM	ICD-10-CM
Major bleeding[‡]	430, 431, 432, 578, 719.1, 423.0, 599.7, 626.2, 626.8, 627.0, 627.1, 786.3, 784.7, 459.0	I60, I61, I62, K92.0, K92.1, K92.2, M25.0, I31.2, R31.0, R31.1, R31.2, R31.9, R04.0, R04.2, R04.81, R04.89, R58, N89.7, N92.0, N92.4, N92.5, N93.8, N95.0
**Censored outcome**		
Atrial fibrillation	427.31	I48.0, I48.2, I48.91

ICD, international classification of diseases; CM, clinical modification

[‡] Major bleeding included intracranial hemorrhage, gastrointestinal bleeding, hemoptysis, subarachnoid hemorrhage, subdural hemorrhage, blood in stool, hemarthrosis, hemopericardium, hematuria and epistaxis)

Supplemental Table S2. International Classification of Diseases, Ninth Revision, Procedure Coding System, International Classification of Diseases, Tenth Revision, Procedure Coding System and National Health Insurance code used for study variables

**Items**	**ICD-9-PCS**	**ICD-10-PCS**	**NHI code**
Coronary artery bypass grafting (CABG)	36.1	0210, 0211, 0212, 0213	
Percutaneous coronary intervention (PCI)	36.01, 36.02, 36.05, 36.06, 36.09, 0.66	02703, 02713, 02723, 02733, 02C03, 02C13, 02C23, 02C33	
Renal transplantation	55.6	0TS00ZZ, 0TS10ZZ, 0TY00Z0, 0TY00Z1, 0TY00Z2, 0TY10Z0, 0TY10Z1, 0TY10Z2	76020A, 76020B, 97416K
Mechanical Valve	35.22, 35.24		
Bioprosthetic Valve	35.21, 35.23		

ICD, international classification of diseases; PCS, procedure coding system; NHI, national health insurance

Supplemental Table S3. The drug code of concomitants in the study

Concomitant drug	ATC code				
ACEI	C09AA01	C09AA04	C09AA07	C09AA16	C09BA04
	C09AA02	C09AA05	C09AA08	C09BA01	C09BB04
	C09AA03	C09AA06	C09AA09	C09BA02	C09BB05
ARB	C09CA01	C09CA06	C09DA01	C09DA08	C09DB04
	C09CA02	C09CA07	C09DA03	C09DA09	C09DX01
	C09CA03	C09CA08	C09DA04	C09DB01	C09DX03
	C09CA04	C09CA09	C09DA07	C09DB02	C09DX04
CCB	C08CA01	C08CA06	C08CA15	C09DB01	C09DX03
	C08CA02	C08CA08	C08DA01	C09DB02	C09XA
	C08CA03	C08CA09	C09BB	C09DB04	C09XA52
	C08CA04	C08CA12	C09BB04	C09DB07	C10BX03
	C08CA05	C08CA13	C09BB05	C09DX01	
beta blocker	C07AA01	C07AA07	C07AB03	C07AB12	C07CA03

Concomitant drug	ATC code				
	C07AA02	C07AA12	C07AB04	C07AG01	
	C07AA03	C07AA15	C07AB05	C07AG02	
	C07AA05	C07AA19	C07AB07	C07BB02	
	C07AA06	C07AB02	C07AB09	C07BB03	
statin	C10AA01	C10AA04	C10AA08	C10BA03	
	C10AA02	C10AA05	C10BA01	C10BA05	
	C10AA03	C10AA07	C10BA02	C10BX03	
NSAID	M01AA	M01AC	M01AE	M01AG	M01AH
	M01AB				
PPI	A02BC				
H2-blocker	A02BA				
digoxin	C01AA				
amiodarone	C01BD01				

CCB, calcium channel blocker; ACEI, angiotensin-converting-enzyme inhibitor; ARB, angiotensin II receptor antagonists; NSAID, non-steroidal anti-inflammatory; PPI, proton pump inhibitors

Supplemental Table S4. The drug code of oral antithrombotic therapies in the study

Drug	ATC code	NIH code						
aspirin	B01AC06	A004813100	A040658100	A0429151G0	A0440161G0	A0551041G0	AC43142100	AC54985100
		A021870100	A040665100	A042934100	A044069100	AC37344100	AC43212100	AC549851G0
		A023330100	A0406651G0	A0429341G0	A044176100	AC373441G0	AC43254100	AC551041G0
		A023534100	A041018100	A043139100	A0441761G0	AC37702100	AC43309100	B023619100
		A024465100	A041073100	A043142100	A044578100	AC41220100	AC433091G0	B023919100
		A025848100	A041220100	A043212100	A045015100	AC41511100	AC43663100	B024025100
		A028936100	A041511100	A043254100	A045072100	AC415111G0	AC436631G0	B0240251G0
		A029740100	A0415111G0	A043309100	A048339100	AC41814100	AC43664100	BC23919100
		A030696100	A041814100	A0433091G0	A0483391G0	AC418141G0	AC436641G0	BC24025100
		A034975100	A0418141G0	A043663100	A048542100	AC42461100	AC44176100	BC240251G0
		A036599100	A042461100	A0436631G0	A0485421G0	AC424611G0	AC441761G0	C001621100
		A037344100	A0424611G0	A043664100	A0495361G0	AC42774100	AC48542100	N004155100
		A0373441G0	A042774100	A0436641G0	A054863100	AC42934100	AC495361G0	N011693100
**Drug**	**ATC code**	**NIH code**						
		A037702100	A042915100	A044016100	A0548631G0	AC43139100	AC548631G0	

Drug	ATC code	NIH code						
clopidogrel	B01AC04	A047589100	A050241100	AA49344100	AB58093100	AC52522100	B025114100	BC25114100
		A048062100	A052522100	AA50126100	AC48062100	AC55026100	B025222100	BC25222100
		A048649100	A055026100	AA57140100	AC48649100	AC55428100	B025326100	BC25326100
		A048730100	A055044100	AB48649100	AC49224100	AC57140100	B026190100	BC25873100
		A049224100	A055428100	AB48730100	AC49344100	AC57819100	B026335100	BC26190100
		A049344100	A057123100	AB49344100	AC49719100	AC58093100	BB25873100	BC26252100
		A049719100	A057140100	AB49719100	AC49967100	B022932100	BC22932100	BC26335100
		A049967100	AA48649100	AB50126100	AC50126100	B024863100	BC24863100	BC27182100
		A050126100	AA48730100	AB57819100	AC50241100	B025034100	BC25034100	
cilostazol	B01AC23	A044124100	A048377100	A050027100	AB44124100	AC44124100	AC48377100	AC50027100
		AC57814100	B023005100					
dipyridamole	B01AC07	A020095100	A025406100	A025431100	A027445100	A028507100	A028592100	A0285921G0
		A029432100	A029766100	A031228100	A031323100	A032046100	A0320461G0	A038495100
		A041877100	A042535100	A043040100	A043630100	AC25406100	AC27445100	AC274451G0
		AC285071G0	AC28592100	AC285921G0	AC294321G0	AC31228100	AC312281G0	AC32046100

		AC320461G0	AC41877100	AC42535100	AC425351G0	AC43040100	AC430401G0	AC43630100
		B008358100	B013590100	B016102100	B016141100	B018442100		
ticlopidine	B01AC05	A033091100	A036756100	A036857100	A039559100	A042830100	AB33091100	AC33091100
		AC42830100	B015133100	B020841100				
Aggrenox (aspirin+ dipyridamole)	B01AC30	BC23919100						
warfarin	B01AA03	A043862100	A052559100	AC50423100	B020346100	B020516100	B023573100	X000140100
		A050095100	AC43862100	AC52559100	B020354100	B023426100	BC23572100	A050423100
		AC50095100	AC55271100	B020515100	B023572100	BC23573100		
dabigatran	B01AE07	B025458100	B025459100	BC25458100	BC25459100	BC26233100		
rivaroxaban	B01AF01	B025129100	B025647100	B025648100	BC25129100	BC25647100	BC25648100	
apixaban	B01AF02	BC26124100	BC26133100					
edoxaban	B01AF03	BC26599100	BC26600100					

Supplemental Table S5. Oral antithrombotic therapies included in the study

Category	Drug ingredient
Antiplatelet	(1) aspirin; (2) clopidogrel; (3) ticlopidine;<br>(4) dipyridamole; (5) cilostazol
Anticoagulant	(1) warfarin; (2) rivaroxaban; (3) dabigatran;<br>(4) apixaban; (5) edoxaban
Combination therapy	(1) aspirin + clopidogrel; (2) aspirin + dypyridamole;<br>(3) aspirin + ticlopidine; (4) aspirin + cilostazol;<br>(5) aspirin + warfarin; (6) aspirin + DOAC;<br>(7) clopidogrel + warfarin; (8) clopidogrel + DOAC;<br>(9) aspirin + clopidogrel + warfarin;<br>(10) aspirin + clopidogrel + DOAC

DOAC, direct oral anticoagulant

Supplemental Table S6. Incidence of study outcomes among APT, OAC and combination group

	Incidence Rate		
	**APT**	**OAC**	**Combination**
Ischemic Stroke	42.88	42.26	81.08
Systemic Embolism	2.46	4.46	25.91
Composite of Stroke	40.61	47.04	109.84
MACE	116.46	85.65	158.68
Major bleeding	57.42	66.66	53.63
All-cause mortality	138.99	48.94	217.06
Cardiovascular-related death	75.59	26.70	127.68

APT, antiplatelet; OAC, oral anticoagulants; MACE, major adverse cardiac event;

*Event divided by 1000 person years

Supplemental Table S7. Subgroup analysis stratified with gender in patients with AFL and CKD

	Adjusted Hazard Ratio					
	APT vs. Combination		OAC vs. Combination		APT vs. OAC	
	95%CI	p-value	95%CI	p-value	95%CI	p-value
**Male**						
Ischemic Stroke	0.61 (0.11-3.47)	0.58	0.32 (0.03-3.82)	0.37	1.34 (0.26-7.06)	0.72
All-cause mortality	0.38 (0.12-1.15)	0.09	0.05 (0.01-0.43)	0.007	2.78 (0.59-13.14)	0.19
**Female**						
Ischemic Stroke	0.61 (0.18-2.03)	0.47	0.72 (0.17-3.17)	0.67	0.67 (0.25-1.87)	0.45
All-cause mortality	1.03 (0.52-1.92)	0.94	0.32 (0.12-0.82)	0.02	2.75 (1.32-5.76)	0.007

APT, antiplatelet; OAC, oral anticoagulants; CI, confidential interval

Adjusted variables included age, congested heat failure, hypertension, diabetes mellitus, vascular diseases, chronic obstructive pulmonary disease, dyslipidemia, gout, abnormal liver function, gastric ulcer and bleeding history.
